# Ochratoxin A and Aflatoxins in Liquorice Products

**DOI:** 10.3390/toxins2040758

**Published:** 2010-04-20

**Authors:** Amedeo Pietri, Silvia Rastelli, Terenzio Bertuzzi

**Affiliations:** Istituto di Scienze degli Alimenti e della Nutrizione, Facoltà di Agraria, Università Cattolica del Sacro Cuore, Via Emilia Parmense, 84 I-29100 Piacenza, Italy; Email: terenzio.bertuzzi@unicatt.it (T.B.)

**Keywords:** ochratoxin, aflatoxins, liquorice

## Abstract

The occurrence of ochratoxin A (OTA) and aflatoxins (AFs) in liquorice products made in Italy was surveyed. Twenty-eight samples of dried liquorice extract and fifty-four of liquorice confectionery (liquorice content between 2 and 10%) were collected from retail outlets located in northern Italy. After extraction and purification through an immunoaffinity column, OTA and AFs were analysed using both HPLC-FLD and HPLC-MS/MS. OTA occurred in all samples of dried liquorice extract and in 61% of samples of liquorice confectionery, showing very high values for the former (mean 89.6 µg kg^-1^, maximum value 990.1 µg kg^-1^), and relatively low levels for the latter (mean 0.96 µg kg^-1^, maximum value 8.3 µg kg^-1^). The contribution of dried liquorice extract to OTA intake appears to be non-negligible for children, who are potentially high consumers. AF contamination resulted very low: AFB_1_ was detected only in 15.8% of samples (maximum value 7.7 µg kg^-1^, mean 0.38 and 0.41 µg kg^-1^ for dried liquorice extract and liquorice confectionery, respectively); the other AFs were not detected. To our knowledge, it is the first time that AFB_1_ has been detected in liquorice extract samples.

## 1. Introduction

Liquorice (*Glycyrrhizia glabra* L.) is a perennial herbaceous plant; it is cultivated mainly in countries of the Mediterranean area, but it is also widespread in Germany, Russia, China and Australia. Liquorice is a widely consumed medicinal herb and a common dietary supplement; it is used either as a fresh or dry rhizome (root), or as a liquorice extract; the latter is obtained after treatment of roots with steam or hot water and following concentration of the extract, to produce a syrup-like material or a solid liquorice block. Liquorice derivatives are widely used for the preparation of confectionery and other food products. 

Ochratoxin A (OTA) is a mycotoxin produced by various *Aspergillus* and *Penicillium* species [1]. Several studies have shown that the toxin has carcinogenic, nephrotoxic, immunotoxic, teratogenic, and possibly neurotoxic and genotoxic properties and it has also been associated with Balcan Endemic Nephropathy in humans [2,3,4,5]. OTA contaminates many foods, such as cereals and derived products, dried fruit, coffee, cocoa, some spices, wine, grape juice, beer and ripened pork products [6,7,8,9].

Aflatoxins (AFs: AFB_1_, AFB_2_, AFG_1_, AFG_2_) are produced primarily by *Aspergillus flavus* and *A. parasiticus* [1]. AFB_1_, the most toxic and widespread of AFs, is a potent genotoxic carcinogen in laboratory animals and there is strong evidence that it is a liver carcinogen in humans [10]. AF contamination concerns mainly dried fruit (nuts, peanuts, pistachio nuts, dried figs), maize and maize products and some spices [11,12,13].

OTA and AFB_1_ have been classified by the International Agency for Research on Cancer (IARC) as a class 2B (possible human carcinogen) and class 1 (human carcinogen), respectively [14,15]. The European Commission (EC) Regulation No 1881/2006 [16] set maximum limits for OTA, AFB_1_ and total aflatoxins in different foods. Recently, the European Commission (EC) Regulation No 105/2010 [17], amending Regulation 1881/2006, fixed maximum levels of 20 and 80 µg kg^-1^ for liquorice root, (ingredient for herbal infusion) and for liquorice extracts (for use in liquorice confectionery), respectively (Table 1); this Regulation shall apply from 1 July 2010. The European Food Safety Authority (EFSA), on a request from the EC, adopted an updated scientific opinion relating to OTA in food and fixed a tolerable weekly intake (TWI) of 120 ng kg^-1^ bw [18]. As regards AFB_1_, a tolerable daily intake (TDI) was not set, because of its genotoxic properties; therefore, contamination in food should be reduced to the lowest possible level. 

The possible presence of OTA in liquorice was pointed out after it was noticed that the toxin occurred in herbal tea, in which liquorice was among the ingredients, but not in black tea [19]. Consequent studies confirmed widespread and high contamination of OTA in foods containing liquorice, sometimes with values exceeding 200 µg kg^-1^ [20,21,22,23,24,25]; on the contrary, no data are available on AF occurrence in liquorice. The selection of liquorice rhizomes based on homogeneity of colour did not result in a significant reduction of OTA contamination; on the contrary, peeling of roots and processing for the production of liquorice extract and block liquorice significantly reduced the OTA level [26]. However, these processes do not eliminate the problem and a non-negligible OTA contamination can remain in liquorice extract and consequently in liquorice-containing confectionery, sweets widely consumed by children.

In a preliminary survey on a limited number of samples (20) purchased in Italy, we found high levels of OTA in dried liquorice products and limited AFB_1_ contamination in dried liquorice products and liquorice confectionery (2–10% of liquorice) [27]. The present study reports the results of a further survey, on the incidence and levels of OTA and AFs in these two types of products and evaluates their contribution to OTA and aflatoxin intake in humans.

**Table 1:** EU maximum admissible limits for OTA, AFB_1_, total aflatoxins (sum of AFB_1_, AFB_2_, AFG_1_, AFG_2_) in foodstuffs for direct human consumption.

	OTA (µg kg^-1^)	AFB_1_ (µg kg^-1^)	Sum of AFB_1_, AFB_2_, AFG_1_ and AFG_2_(µg kg^-1^)
Cereals for direct human consumption	3.0	2.0	4.0
Groundnuts, nuts and dried fruit for direct human consumption	-	2.0	4.0
Spices (*Capsicum* spp., *Piper* spp., *Myristica fragrans*, *Zingiber officinale*, *Curcuma longa*)	30^*^	5.0	10.0
Dried wine fruit	10.0	-	-
Wine and grape juice	2.0	-	-
Roasted coffee	5.0	-	-
Soluble coffee	10.0	-	-
Processed cereal-based foods and baby foods for infants and young children	0.50	0.1	
Liquorice root	20		
Liquorice extract	80		

^* ^15 µg kg^-1^ as from 01.7.2012.

## 2. Results and Discussion

### 2.1. Performance of the analytical method

The samples were initially analysed, both for OTA and for AFs, using HPLC with fluorimetric detection (HPLC-FLD); however, accurate identification and quantification was not possible in some extracts, because of the presence of interfering peaks near those of OTA and AFB_1_. Therefore, the samples were analyzed by HPLC coupled with mass spectrometry (HPLC-MS/MS). The recovery values were estimated by spiking a blank sample with appropriate volumes of OTA and AF standards, in order to have contamination levels of 2 and 10 µg kg^-1^ for OTA and for each AF. Average recovery values, obtained by HPLC-MS/MS analysis, were above 89% for OTA and AFB_1_ (Table 2), and above 86% for the other AFs. 

The limits of detection (LOD) and of quantification (LOQ) were defined at those levels resulting in signal-to-noise ratios of 3 and 10, respectively. The analyte response and the chromatographic noise were both measured from the chromatogram of a blank sample extract (0.5 mL), to which volumes between 0.1 and 0.5 mL of OTA and AFs solutions (0.066 and 0.115 µg L^-1^, respectively) had been added. The LOD and LOQ values were 0.12 and 0.35 µg kg^-1^ for OTA, 0.25 and 0.60 µg kg^-1^ for each of the four AFs. The calibration curve showed good linearity over the range 0.2–20 pg for OTA and 0.25–2 pg for AFs (r > 0.998). To test for matrix effect, a second calibration curve was generated from standard solutions prepared by dilution with blank purified extract of dried liquorice extract and liquorice confectionery. The 95% confidence intervals for the slopes and y-intercepts of the two curves overlapped, indicating no significant matrix effect.

**Table 2:** Recovery of OTA and AFB_1_ from artificially contaminated liquorice products.

			Recovery (%)	
	**Spike level (µg kg^-1^)**	**Number of analyses**	**Mean ± standard deviation**	**Range**
*OTA*				
Dried liquorice extract	2	4	92.2 ± 1.7	90.1–94.2
	10	4	91.8 ± 1.5	90.0–93.6
Liquorice confectionery	2	4	91.0 ± 1.8	89.1–93.3
	10	4	90.6 ± 1.6	88.7–92.5
*AFB_1_*				
Dried liquorice extract	2	4	90.6 ± 1.8	88.4–92.5
	10	4	90.9 ± 1.6	89.0–92.8
Liquorice confectionery	2	4	89.7 ± 2.3	87.5–91.8
	10	4	89.5 ± 2.0	87.3–91.5

**Figure 1:**
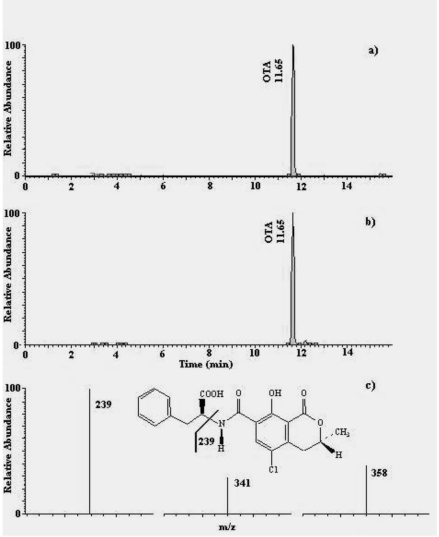
Chromatograms (HPLC-MS/MS) of: (a) an OTA standard solution (5.06 µg L^-1^), equivalent to 25.3 pg of OTA injected; (b) a naturally-contaminated dried liquorice extract sample containing 136.9 µg kg^-1^ (equivalent to 34.2 pg of OTA injected); (c) selected fragment ions of OTA, derived from the 404 *m/ z* parent ion.

**Figure 2:**
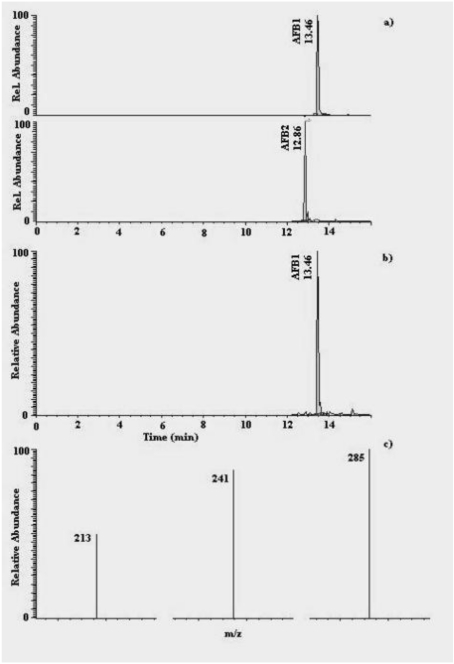
Chromatograms (HPLC-MS/MS) of: (a) an AFB_1_ and AFB_2_ standard solution (0.93 μg L^-1^ for each AF), equivalent to 4.6 pg of AFB_1_ and AFB_2 _injected; (b) a naturally-contaminated dried liquorice extract sample containing 2.19 µg kg^-1^ (equivalent to 0.55 pg of AFB_1_ injected); (c) selected fragment ions of AFB_1_ derived from the 313 *m/z* parent ion.

Concerning HPLC-MS/MS analysis, performance criteria fixed by Decision 2002/657/EC [28] were fulfilled completely. Figure 1 and Figure 2 show HPLC-MS/MS chromatograms and the selected fragment ions of a standard solution and a sample of naturally-contaminated liquorice product, for OTA and AFB_1_, respectively. As regards OTA, the formation of the ions at 358 and 341 are explained by the loss of H_2_O + CO (358 *m/z*) and OH (341 *m/z*); the product ion at 239 *m/z* results from molecule cleavage as shown in Figure 1 [20,29]. As regards AFB_1_, formation of the ions was in agreement with previous works [30,31,32]. 

For HPLC-FLD analysis of OTA, LOD and LOQ were 0.05 and 0.15 µg kg^-1^, respectively; the calibration curve showed good linearity over the range 0.1–20 pg (r > 0.998). 

The results, corrected for recovery, are reported in Table 3.

### 2.2. Ochratoxin A

OTA was present in 61 samples, with an incidence of 74.4%; among these, 47 (57.3%) showed an OTA concentration higher than the LOQ. A good correlation between the results obtained by HPLC-FLD and by HPLC-MS/MS was observed at concentrations exceeding 1.0 µg kg^-1^ (r = 0.98). At lower levels HPLC-MS/MS did not always confirm the identity of the peak detected by HPLC-FLD. In fact, in 11 samples (13.4%) showing an OTA contamination in the range 0.35–1 µg kg^-1^ if measured by HPLC-FLD, the OTA presence was not confirmed by HPLC-MS/MS; in another eight samples (9.7%), lower levels were found. 

With regard to dried liquorice extract samples, OTA was always detected, also at high concentrations. The incidence of samples with an OTA contamination level higher than 10 µg kg^-1^ was 71.4%; among these, five samples (17.8%) showed a concentration higher than 80 µg kg^-1^ (87.1, 113.4, 148.8, 600.5 and 990.1 µg kg^-1^), the limit for liquorice extract fixed by the Commission Regulation No 105/2010 [17]. Our data confirmed the widespread and high OTA contamination observed in previous surveys [20,21,22,23,24,25]. Bresch *et al.* [20] detected OTA in about 50% of liquorice roots and in almost all the products (95%) obtained after the extraction and concentration process; Arino *et al.* [21] and Majerus *et al.* [22] found the toxin in all the samples of dried liquorice extract. In our survey the maximum value was similar to that found in a liquorice root sample (905 µg kg^-1^) by Kabelitz and Sievers [25].

**Table 3:** Occurrence of OTA and AFB_1_ in dried liquorice extracts and liquorice confectionery.

	n	Positives > LOD (>LOQ)	Incidence of positives (%)	Mean of positives (µg kg^-1^)	Mean of all^*^ (µg kg^-1^)	Median (µg kg^-1^)	Maximum value (µg kg^-1^)
*OTA*							
Dried liquorice extract	28	28 (27)	100	89.6	89.6	26.3	990.1
Liquorice confectionery	54	33 (20)	61.1	1.53	0.96	0.17	8.3
*AFB_1_*							
Dried liquorice extract	28	5 (4)	17.9	1.57	0.38	<LOD	2.4
Liquorice confectionery	54	8 (5)	14.8	2.06	0.41	<LOD	7.7

^*^ Mean value was calculated assuming a value of ½ LOD for samples <LOD.

Regarding liquorice confectionery products, the contamination was not negligible, as 11 (20.4%) and four (7.4%) samples showed a concentration higher than 1 and 4 µg kg^-1^, respectively; the latter could be a possible maximum value for OTA, considering the maximum limit of 80 µg kg^-1^ and a 5% mean content of liquorice extract in liquorice confectionery products. In previous surveys, Majerus *et al.* and Bresch *et al.* detected OTA in 24 out of 26 and 18 out of 19 samples of liquorice confectionery sweets respectively (incidence of positives samples > 90%); Herrera *et al.* [24] detected OTA in 75 and 39% of samples of hard and soft candies, respectively. The mean value in this survey (0.96 µg kg^-1^) resulted similar to those obtained by Majerus *et al.*(1.1 µg kg^-1^), Bresch *et al.* (1.3 µg kg^-1^) and Herrera *et al.*(1.29 µg kg^-1^).

### 2.3. Aflatoxins

Only AFB_1_ was detected; the contamination resulted very low both for dried liquorice extract and for liquorice confectionery samples. Thirteen samples (15.8%) showed values higher than 0.25 µg kg^-1^, the LOQ for the HPLC-MS/MS method. In another four samples (4.9%), AFB_1 _was detected by HPLC-FLD (LOD 0.1 µg kg^-1^) at lower levels. The maximum value was 7.7 µg kg^-1^ (liquorice confectionery sample) and only in three samples was the concentration higher than 2 µg kg^-1^ (EC limit for AFB_1_ in maize and dried fruit). As regards confectionery products, the low level of AFB_1_, found in few samples, could be also due to other ingredients. 

### 2.4. Estimation of OTA dietary intakes by the consumption of liquorice products

Because of the widespread contamination with OTA, it is of interest to estimate the daily average intake for liquorice products, even if they are not a staple food; however, they are widely consumed, mainly by children. As the contamination levels (median values) in dried liquorice extract and in liquorice confectionery samples were very different, the average daily OTA intake was calculated for both products. Liquorice improves overall health and alleviates a wide range of diseases [33], but its ingestion should be limited for the presence of glycyrrhizinic acid, which, at amounts exceeding 100 mg day^-1^, can cause different ailments, such as retention of urine and high blood pressure; moreover, the ingestion of liquorice products is contraindicated for diabetics and/or those with high blood pressure [34,35]. In dried liquorice extract, glycyrrhizinic acid ranges between 4 and 25%, so it is recommended to eat no more than 0.75 g day^-1^ of these products (about 2–3 sweets). Regarding liquorice confectionery, the European Commission carried out a study on north-European populations, that estimated an average daily consumption per person (consumers and non consumers), from 2.7 to 6.8 g; the intake increased to 11.5 g for high consuming children [36]. Considering the median values of the present survey (26.3 and 0.17 µg kg^-1^ for dried liquorice extract and liquorice confectionery samples, respectively) and an average consumption of 0.75 and 6.8 g day^-1^, it was calculated an OTA daily intake of 19.7 and 1.1 ng for dried liquorice extract and liquorice confectionery samples, respectively (corresponding to a weekly intake of 137.9 and 7.7 ng). The European Food Safety Authority (EFSA) derived a tolerable weekly intake (TWI) for OTA of 120 ng kg^-1 ^bw [18]; considering a body weight of 70 and 30 kg for adults and children, respectively, the weekly average exposure for dried liquorice extract resulted equivalent to 1.6 and 3.8% of the TWI, while for liquorice confectionery to 0.1 and 0.2%. These values indicated that exposure from liquorice confectionery was negligible, in agreement with the study of Herrera *et al.* [24], which reported values of 0.13 and 0.31% of the TWI for adults and children, respectively. On the contrary, for a child regularly consuming dried liquorice extract sweets, the intake is not negligible, considering that OTA can be present in many other foods in the diet.

## 3. Experimental Section

### 3.1. Samples

In years 2006 and 2007, 28 samples of dried extract liquorice and 54 samples of liquorice confectionery (2–10% of liquorice) were collected from retail outlets in northern Italy; all the products were made in Italy. For each sample, an aliquot of 100 g or the whole package if the amount was lower, was frozen at –20 °C and rapidly milled using a coffee-grinder; then, the sample was homogenized, immediately frozen again and kept at –20 °C until the time of analysis. All samples were analysed using both HPLC-FLD and HPLC-MS/MS. 

### 3.2. Reagents and standards

HPLC grade acetonitrile, methanol and acetic acid were purchased from Merck (Darmstadt, Germany). Ultrapure water was obtained from a Milli-Q apparatus from Millipore (Milford, MA, USA). The immunoaffinity columns for OTA and AFs were supplied by R-Biopharm Rhône LTD (Glasgow, Scotland, UK). Phosphate buffered saline (PBS) was prepared as per R-Biopharm Rhône LTD (NaCl 8 g L^-1^, KCl 0.2 g L^-1^, Na_2_HPO_4_ 1.15 g L^-1^, KH_2_PO_4_ 0.2 g L^-1^; pH 7.4).

OTA and AFs standards were obtained from Sigma-Aldrich (St. Louis, MO, USA). A solution of OTA (40 µg mL^-1^ in benzene-acetic acid 99:1) was calibrated spectrophotometrically at 333 nm using the value 5,550 L mol^-1 ^cm^-1^ for the absorption coefficient [37] and stored at –20 °C when not in use; working standards were prepared by evaporating an exact volume under a stream of nitrogen and re-dissolving the residue in the mobile phase. Regarding HPLC-MS/MS, eight OTA standards in the range between 0.04 and 4 µg L^-1^ were injected. For each AF, a stock solution of 5–8 µg mL^-1^ was prepared in benzene:acetonitrile (98:2 v/v, 2 mL) and stored at –20 °C. The solutions were calibrated spectrophotometrically at 350 nm, using the values 19,800, 20,900, 17,100 and 18,200 L mol^-1 ^cm^-1^ for the absorption coefficient of AFB_1_, AFB_2_, AFG_1_ and AFG_2_, respectively [38]; the solutions were stored at –20 °C when not in use; working standards were prepared by evaporating an exact volume under a stream of nitrogen and re-dissolving the residue in the mobile phase. Regarding HPLC-MS/MS, eight calibrant solutions at individual concentrations of AFB_1_, AFB_2_, AFG_1_, AFG_2_ between 0.05 and 0.4 µg L^-1 ^were injected.

### 3.3. Analysis for OTA

OTA was extracted from a 10 g portion of sample, with 100 mL of a mixture of sodium bicarbonate 0.13M-methanol (50 + 50 v/v) for 45 min using a rotary-shaking stirrer. After filtration through a folded filter paper, an aliquot of the filtrate (5 mL) was diluted with PBS (50 mL) and purified through an immunoaffinity column (Ochraprep, R-Biopharm Rhône LTD). The column was washed with PBS (2 mL) and OTA was slowly eluted (0.5 mL min^-1^) with methanol acidified with acetic acid (98 + 2 v/v, 2.5 mL) into a graduated glass vial: the eluate was concentrated under a gentle stream of nitrogen, brought to 1 mL with acetonitrile:2% acetic acid (41 + 59 v/v) and vortex-mixed for few seconds. The extract was filtered (HV 0.45 µm, Millipore Corporation, Bedford, Massachusetts, USA) before HPLC analysis. 

#### 3.3.1. HPLC-FLD analysis

The HPLC system consisted of a Perkin Elmer 200 (Perkin Elmer, Norwalk, Connecticut, USA), equipped with a Jasco AS 1555 sampling system and a FP 1520 fluorescence detector (Jasco Corporation, Tokyo, Japan) set at 333 nm excitation and 470 nm emission wavelength. The system was governed by a Borwin 1.5 software (Jasco). A RP-18 column (4 µm particle size, 125 × 4 mm i.d., Merck) was employed at ambient temperature, with a mobile phase of acetonitrile:2% acetic acid (41 + 59 v/v) at 1.0 mL min^-1^. The injection volume was 30 µL.

#### 3.3.2. HPLC-MS/MS analysis

The identification and quantification of OTA were carried out by HPLC-MS/MS analysis. The HPLC-MS/MS system consisted of a LC 1.4 Surveyor pump (Thermo Fisher Scientific, San Jose, CA, USA), a PAL 1.3.1 sampling system (CTC Analitycs AG, Zwingen, Switzerland) and a Quantum Discovery Max triple quadrupole mass spectrometer; the system was controlled by a Excalibur 1.4 software (Thermo-Fisher). After dilution of the purified extracts (0.1 mL brought to 0.5 mL) with acetonitrile:water (60 + 40 v/v, acidified with 0.4% acetic acid), OTA was chromatographed on a Betasil RP-18 column (5 µm particle size, 150 × 2.1 mm i.d., Thermo-Fisher) and separated using gradient elution with acetonitrile and water as mobile phase A and B, respectively (both acidified with 0.4% acetic acid). The gradient program was as follows: at time zero 40% solvent A; linear gradient to 75% solvent A within 8 min, then isocratic for 5 min. The flow rate was 0.2 mL min^-1^. The ionization was carried out with an ESI interface (Thermo-Fisher) in positive mode as follows: spray capillary voltage was 4.0 kV, sheath gas and auxiliary gas 30 and 5 psi, respectively; temperature of the heated capillary 270 °C. The mass spectrometric analysis was performed in selected reaction monitoring (SRM). For fragmentation of [M + H]^+^ ions (404 *m/z*), the argon collision pressure was 1.5 mTorr and the collision energy was 25 and 15 V. The detected and quantified fragment ions were 358, 341 and 239 *m/z*. Quantitative determination was performed by a LC-Quan 2.0 software (Thermo-Fisher). 

### 3.4. Analysis for AFs

AFs were extracted from a 10 g portion of sample with 100 mL acetone-water (70 + 30 v/v) using a rotary-shaking stirrer for 45 min. After filtration through a folded filter-paper, an aliquot of the filtrate (5 mL) was diluted with distilled water (45 mL) and the solution was purified through an immunoaffinity column (Easi-Extract Aflatoxin, R-Biopharm Rhône LTD). After washing the column with 5 mL distilled water, AFs were eluted into a graduated glass vial with methanol (2.5 mL). The eluate, concentrated under a gentle stream of nitrogen, was brought to 1 mL with acetonitrile:water (25 + 75 v/v) and vortex-mixed for few seconds; then, the extract was filtered (HV 0.45 µm, Millipore), before HPLC analysis. 

#### 3.4.1. HPLC-FLD analysis

Analysis was performed using an HPLC instrument, consisting of two PU-1580 chromatographic pumps, an AS 1555 sampling system, a FP 1520 fluorescence detector set at 365 nm excitation and 440 nm emission wavelength, and a post-column derivatization system (Jasco); the instrument was governed by a Borwin 1.5 software (Jasco). A Superspher RP-18 column (4 μm particle size, 125 × 4 mm i.d., Merck) was used at ambient temperature, with a mobile phase of water:methanol:acetonitrile (64:23:13 v/v/v), at 1.0 mL min^-1^. A solution of pyridinium bromide perbromide (25 mg in 500 mL of HPLC-grade water) was used as derivatizing reagent; the flow of the solution was set at 0.1 mL min^-1^ and the volume of the reaction tube was 500 µL. The injection volume was 30 µL.

#### 3.4.2. HPLC-MS/MS analysis

The identity and quantification of AFs were carried out by HPLC-MS/MS analysis. The HPLC-MS/MS system consisted of a LC 1.4 Surveyor pump (Thermo Fisher Scientific), a PAL 1.3.1 sampling system (CTC Analytics AG) and a Quantum Discovery Max triple quadrupole mass spectrometer; the system was controlled by an Excalibur 1.4 software (Thermo-Fisher). After dilution of the purified extracts (0.1 mL brought to 0.5 mL) with acetonitrile-water (60 + 40 v/v, acidified with 0.4% acetic acid), AFs were chromatographed on a Betasil RP-18 column (5 µm particle size, 150 × 2.1 mm i.d., Thermo-Fisher) and separated using gradient elution with acetonitrile and water as mobile phase A and B, respectively (both acidified with 0.4% acetic acid). The gradient program was as follows: at time zero 15% solvent A; linear gradient to 65% solvent A within 9 min, then isocratic for 5 min. The flow rate was 0.2 mL min^-1^. The ionization was carried out with an ESI interface (Thermo-Fisher) in positive mode as follows: spray capillary voltage was 4.0 kV; sheath gas and auxiliary gas 41 and 5 psi, respectively; temperature of the heated capillary 270 °C. The mass spectrometric analysis was performed in SRM. For fragmentation of [M + H]^+^ ions (313 *m/z*), the argon collision pressure was 1.5 mTorr and the collision energy was 33 and 15 V. The detected and quantified fragment ions were 285, 241 and 213 *m/z*. Quantitative determination was performed by a LC-Quan 2.0 software (Thermo-Fisher). 

## 4. Conclusions

On the basis of the results obtained in this survey, the OTA levels in liquorice extract should be continuously monitored, because of the widespread contamination of this product that leads to a non- negligible OTA presence in liquorice-based confectionery products. There is a need to investigate the origins of this contamination and to apply Good Agricultural Practice principles to cultivation, harvesting and drying of the liquorice roots. With regard to AFs, to our knowledge it is the first time that AFB_1_ has been detected in liquorice extract samples. Here again, there is a need to investigate the origin of this contamination and the possible presence of the toxin in liquorice root used as an ingredient for herbal infusion. 
